# An Integrated Coproscopic and Molecular Method Provides Insights into the Epidemiology of Zoonotic Intestinal Helminths of Dogs across Cambodia

**DOI:** 10.1155/2023/2001871

**Published:** 2023-08-22

**Authors:** Patsy A. Zendejas-Heredia, Vito Colella, Lucas G. Huggins, Roland Schaper, Bettina Schunack, Rebecca J. Traub

**Affiliations:** ^1^Faculty of Science, University of Melbourne, Parkville, Victoria, Australia; ^2^Bayer Animal Health GmbH An Elanco Company, Monheim, Germany

## Abstract

**Introduction:**

In Cambodia, a limited number of focal surveys targeting dogs in rural communities have reported intestinal helminths of potential zoonotic risk as highly endemic. In this study, we investigated the prevalence, diversity, distribution, and risk predictors associated with zoonotic intestinal helminths infecting pet, community, and pagoda dogs across rural and urban settings in Cambodia through an integrated coproscopic and molecular approach.

**Methods:**

Faecal samples, demographic, and clinical data were collected from 457 dogs across Cambodia. Faeces were screened for gastroenteric parasites using sodium nitrate faecal floatation (1.30 SpGr) and multiplex TaqMan-based quantitative polymerase chain reaction (qPCR) assays for differentiation of canine hookworm species (*Ancylostoma ceylanicum*, *Ancylostoma braziliense*, *Ancylostoma caninum*, and *Uncinaria stenocephala*) and for *Strongyloides* spp. Conventional polymerase chain reaction (PCR) and DNA sequencing was used to further characterise eggs resembling zoonotic helminths that could not be designated to a species level by light microscopy alone. In addition, infection intensities for hookworms and *Toxocara* as eggs per gram of faeces were estimated to assess the dog age-dependent contribution of these zoonotic parasites in the environment. Finally, multiple logistic regression analyses were performed to identify risk predictors for gastrointestinal parasitoses.

**Results:**

Using combined coproscopic and molecular methods, we detected at least one helminth in 79% of the sampled dogs. Of these, 78.8% were infected with at least one zoonotic species, with *A. ceylanicum* (50%) and *A. caninum* (50%) constituting the most common parasites followed by *A. braziliense* (20%), *Toxocara canis* (15%), *Strongyloides* spp. (5%), *Dipylidium caninum* (2%), and *Eucoleus* spp. (2%). The indirect zoonotic helminths *Spirometra erinaceieuropaei*, *Spirometra mansoni*, and *Haplorchis yokogawai* contributed to 14% of the total infections in dogs. Dogs were also found mechanically passing eggs of large trematodes such as *Fischoederius elongatus*, *Schistosoma* spp., Paramphistomatidae, and *Gastrothylax crumenifer*. Contradictory to previous reports, the prevalence of *T. canis* was highest in adult dogs with egg shedding intensity peaking in dogs more than 7 years of age. Finally, we found that pale mucous membranes and low total protein were predictors of hookworm infection in dogs (*p* < 0.05).

**Conclusion:**

Dogs within both rural and urban settings across Cambodia are highly infected with a large diversity of zoonotic gastroenteric helminths. This study reports the presence of *A. braziliense*, the aetiological agent of hookworm-related “creeping eruptions” for the first time in Cambodia. Moreover, infection intensity data suggest that adult dogs should also be targeted by deworming campaigns to reduce the prevalence of zoonotic hookworm and *T. canis* infections within the country. These results highlight the need for an integrated approach to canine population management and parasite control in dogs across urban and rural settings in Cambodia to mitigate the public health risks and impacts posed by these helminths.

## 1. Introduction

Intestinal helminths are highly endemic in the tropics and more frequently infect dogs and humans living under conditions of poor hygiene and sanitation [1]. Dogs play a critical role in the transmission of a number of zoonotic parasites, some of which can infect humans as definitive (e.g., *Ancylostoma ceylanicum*) or accidental end-stage host (e.g., *Toxocara canis* and *Ancylostoma braziliense*) [2]. Environmental contamination with intestinal helminth eggs occurs through open defecation. As a consequence, hosts become infected via accidental ingestion of infective parasitic eggs, by species such as, *Toxocara canis and Ancylostoma caninum*, or via penetration of filariform larvae into the skin from contaminated soil or water by *Strongyloides* spp. and/or hookworms [3]. For other helminths, ingestion of raw or undercooked intermediate or paratenic hosts constitutes the primary source of infection (e.g., fish-borne trematodes and food-borne cestodes) [4, 5]. Zoonotic helminths constitute a significant public health burden worldwide due to the high risk of transmission facilitated by uncontrolled dog populations, poor personal, and environmental hygiene, close human–dog interactions, a lack of adequate veterinary care and an absence of appropriate control strategies [6–8]. This holds particularly true for some regions of Southeast Asia (SEA) [1, 8] where environmental contamination with helminth eggs is abundant.

Within -SEA, Cambodia is located in the southern part of the Indochina Peninsula and is divided into 24 provinces [9]. It is home to an estimated 5.9 million children/teenagers <17 years of age and ∼5 million domestic and community dogs, which is likely a gross underestimate [10, 11]. These dogs live in close proximity to people, in particular demographic groups such as children living in rural settings and monks living in pagodas in urban settings [9]. Cambodia borders Vietnam, Laos, and Thailand, which are connected by the great Mekong River delta, and where occurrence of direct, soil-transmitted, and food-borne zoonotic helminth infections are well documented [1, 7, 12, 13]. In Cambodia, infection with the zoonotic helminths *A. ceylanicum* and *Strongyloides stercoralis* have been demonstrated as highly endemic in dogs and humans living in rural villages [14–17].

In dogs, hookworm infection can result in anaemia, haemorrhagic diarrhoea, hypoproteinaemia, and in puppies, potentially death, whereas *S. stercoralis* and *T. canis* can both result in diarrhoea, malnutrition, stunting, and in severe cases, death owing to hyperinfection syndrome or intestinal obstruction, respectively [18]. *Ancylostoma ceylanicum* is the only canine and feline hookworm proven to cause patent infection in humans, at times with accompanying diarrhoea, melena, anaemia, and peripheral eosinophilia and has emerged as the second most common hookworm affecting people in SEA [16, 19–23]. All canine hookworms can cause cutaneous larvae migrans in humans that most commonly manifests as a papular rash that self-resolves within days [24]. However, *A. braziliense* is the primary aetiological agent of “creeping eruptions”, pruritic mobile serpiginous lesions that can take months to resolve in the absence of treatment [24]. In addition to cutaneous lesions, *A. caninum* also induces non-patenteosinophilic enteritis and aphthous ileitis in humans [24–29]. Aside from hookworms, *S. stercoralis* is a largely underdiagnosed parasite with the potential to infect humans [30–32]. Human infection with this parasite can result in an endogenous autoinfection that generates long-lasting clinical signs ranging from unspecific gastrointestinal symptoms to fatal health outcomes in immunosuppressed people [33]. Similarly, *T. canis*, the causative agent of visceral and ocular larva migrans, neurotoxocarosis, and covert toxocarosis in humans [34], is a widespread socioeconomically important neglected parasite [35–37] that affects millions of children in the tropics and subtropics [38].

Previous epidemiological surveys in Cambodia have applied mostly traditional copro-diagnostic techniques and reported a high prevalence of intestinal parasites infecting children and animals, alongside the related consequences these infections have on their health and well-being [14, 17, 39–44]. Although simple, inexpensive, and thus recommended in low-resource settings, these techniques are less sensitive and more labour intensive [45, 46]. In addition, some parasite species shed larvae that do not float, or display morphologically similar eggs that preclude their accurate identification to a species level, potentially meaning that zoonotic risk cannot be assessed in such contexts [24, 47]. In contrast, polymerase chain reaction (PCR)-based techniques have shown to be more sensitive and robust, allowing species-specific detection of mixed anthroponotic and zoonotic parasites in human and animal samples [48–51]. Their application in field surveys has informed the emerging distribution and geographical expansion of zoonotic agents such as *A. braziliense* and *A. ceylanicum* from areas where these parasites were previously thought to be absent [23, 45, 46, 52–55].

Despite the known significance of zoonotic infections in SEA, the contribution of dogs to the burden of human helminth infection is still poorly understood [1]. In this study, we assessed the prevalence, diversity, distribution, and associated predictors of intestinal parasitic infections affecting pet and community dogs across Cambodia with the aid of integrated coproscopic and molecular methods.

## 2. Methods

### 2.1. Study Site

**Figure 1:**
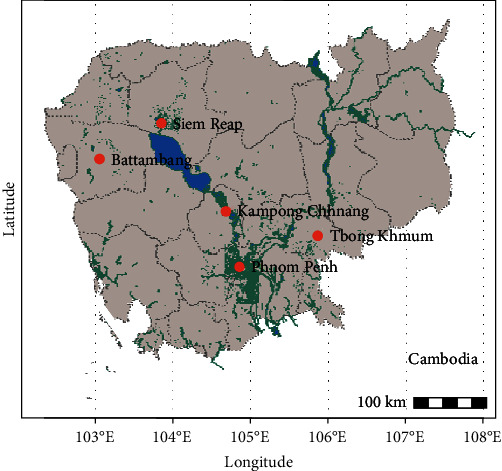
Map of field sites studied, and relative number of samples collected in Phnom Penh (PP; *n* = 100), Siem Reap (SR; *n* = 125), Kampong Chhnang Province (KC; *n* = 39), Battambang province (BB; *n* = 99), and Tbong Khmum district (TB; *n* = 94).

A cross-sectional study was conducted across four provinces and one municipality in Cambodia. Samples were collected in the months of April, May, and September 2019. In Cambodia, the rainy season spans from mid-May to early October. Temperatures range from 28°C in January to 35°C in April, with annual precipitation ranging from 5,000 mm on the seaward slopes to 1,270 mm in the lowland region [9]. Study areas covered were Phnom Penh (PP), Siem Reap (SR), Kampong Chhnang Province (KC), Battambang Province (BB), and Tbong Khmum district (TK) (Figure 1). Study sites were selected to achieve a diversity of urban and rural settings.

### 2.2. Sample Collection

A required sample size of 384 dogs was calculated using the formula *n* = *z*^2^*p*(1−*p*)/*d*^2^, where *n* is the required sample size, *z* (1.96) is the standard deviation at 95% CI, *p* is the expected prevalence (50%), and *d* is the allowed relative error corresponding to the effect size (0.05) [56]. Dogs sampled were free-roaming dogs from a mixture of locally owned and pagoda community dogs cared for by monks. Faecal samples were only taken following informed consent from the relevant monk in the case of pagoda dogs or the owner in case of locally owned dogs. Demographic data were recorded for each animal including age, sex, neutering status, suckling status, husbandry, and location (Table 1). Except for severely unwell dogs, there were no exclusion criteria for the dogs sampled. All the dogs were subjected to a basic physical examination by a qualified veterinarian, and health parameters, that is, rectal temperature, body score, demeanour, haircoat, packed cell volume, total protein, presence of blood and/or mucus in faeces, faecal consistency, and mucous membrane colour (to assess for anaemia, icterus, and hyperaemia), were evaluated and recorded. An abdominal palpation was also conducted alongside assessment of lymph node enlargement. Dog faecal samples were taken directly from the rectal ampulla using a glove and lubricant ensuring the animal was subjected to minimal stress and discomfort. Faecal samples were immediately homogenized in 5% w/v potassium dichromate and kept on ice till refrigerated at 4°C. Samples were then air-transported to the Faculty of Veterinary and Agricultural Sciences, University of Melbourne, on ice and kept refrigerated at 4°C until subjected to coproscopy and molecular analyses.

### 2.3. Faecal Floatation Technique

Faecal samples were subjected to double cover slip centrifugal faecal floatation using sodium nitrate (NaNO_3_, specific gravity (S.G) 1.3) [45, 46], for the observation of helminth ova. Egg counts were performed for hookworms and *Toxocara* spp. with infection intensities calculated in eggs per gram (EPG) of faeces [45, 46].

### 2.4. DNA Extraction and Quantitative Polymerase Chain Reaction (qPCR)

Faecal samples (200 mg each) were subjected to genomic DNA extraction using a Maxwell RSC PureFood GMO and Authentication Kit (Promega Corporation, US) as per the manufacturer's instructions with modifications consisting of an additional bead-beating step performed using 400 *μ*L CTAB buffer and 0.5 mm zirconia/silica beads (Daintree Scientific, AUS) on a FastPrep-24 5G Instrument (MP Biomedicals). After bead-beating and cell lysis, DNA purification was performed using a Maxwell RSC 48 Instrument (Promega). Final eluted DNA (100 *μ*L) was stored at −20°C until further analyses. Extracted DNA was subjected to multiplex real-time PCR (M-qPCR) assays for the detection of hookworm species [51] and *Strongyloides* spp. [57] using equine herpes virus as an independent reaction control as well as a mammalian target used as a DNA extraction control (Table 2).

### 2.5. Conventional PCR and Sanger Sequencing

Samples found positive for potentially zoonotic helminth eggs using faecal floatation that could not be assigned to a species level were subjected to conventional PCR (cPCR) and Sanger sequencing for further characterisation using previously described protocols for the detection of fish-borne trematodes and cestodes, *Eucoleus*-like eggs and *Toxocara* spp. [59–62]. All cPCRs were conducted using HotStartTaq Plus DNA polymerase (Qiagen, DEU) using a SimpliAMP Thermal Cycler (Thermo Fisher Scientific, US). PCR products were run on a 1.5% (w/v) agarose TBE gel containing GelRed nucleic acid stain (Gene Target Solutions, AUS) and those visualised as a clear single amplicon of the correct size, were purified using ExoSAP-IT PCR Product Cleanup Reagent (Thermo Fisher Scientific, USA) and shipped to Macrogen in South Korea for Sanger sequencing. Resulting chromatograms were cleaned using Geneious Prime® 2021.2.2 Java Version 11.0.11+9 and the resulting nucleotide sequences analysed for nucleotide similarity with those in the GenBank database using BLASTn.

### 2.6. Data Analysis

Prevalence data were analysed and visualised in RStudio Team (2020) Integrated Development Environment for R (RStudio, PBC, Boston, MA) and Microsoft Excel 2021. Kappa statistics were applied to assess the agreement between qPCR and coproscopy for the detection of hookworms. The coefficient was considered worse than random if (Ƙ) < 0.00, slight if 0.00 ≤ ƙ ≤ 0.20, fair if 0.21 ≤ ƙ ≤ 0.40, moderate if 0.41 ≤ƙ ≤ 0.60, substantial if 0.61 ≤ƙ ≤ 0.80, and almost perfect if ƙ > 0.80. The 95% confidence intervals were calculated using the Wald method [63].

Descriptive statistics were conducted to describe the distribution of the EPG data and to obtain the geometric mean and their respective 95% confidence intervals. One-way analysis of variance (ANOVA) tests were used to compare the overall EPG mean of each age class with a *p*-value of 0.05 required for significance. When the overall test was significant, a post hoc test using Tukey's multiple comparison was utilized with a single pooled variance between EPG means to assess the level or group of difference for which a significant difference was observed.

Multiple logistic regression analyses were performed in GraphPad Prism version 8.0 (GraphPad Software) to assess the association between positivity to at least one gastrointestinal (GI) parasite group and individually for *Toxocara* spp. or hookworms, with the above-mentioned demographic data and clinical signs. An iterative backward elimination of variables that were not significant were eliminated until the final multiple logistic model was obtained. Associations were considered statistically significant if *p*  < 0.05. The best-fit model was selected using Akaike's corrected Information Criterion value, multicollinearity was checked using variance inflation factors to check for strongly dependent predictors. Area under the receiver operating characteristic curve (ROC) was used to measure the ability of the model to classify negative and positive events.

## 3. Results

### 3.1. Prevalence of Gastrointestinal Parasites in Dogs

**Figure 2:**
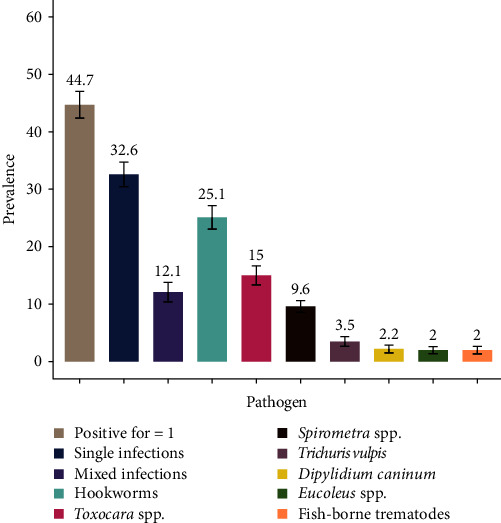
Overall prevalence of gastrointestinal parasites in dogs from five localities in Cambodia using sodium nitrate faecal floatation (S.G 1.3). Error bars represent the standard error of the mean.

Single faecal samples were collected from a total of 457 dogs from five locations: PP *n* = 100, SR *n* = 125, KC *n* = 39, BB *n* = 99, and TB *n* = 94. Seven taxa of intestinal helminths were identified by microscopy infecting 44.7% (207/457, 95% CI: 40.1–49.3) of the sampled dogs. Hookworm eggs were most commonly detected in 25.1% (115/457, 95% CI: 21.1–29.1) of canine faecal samples followed by *Toxocara* spp. in 15% (67/457, 95% CI: 11.4–17.9), *Spirometra* eggs in 9.6% (44/457, 95% CI: 6.9–12.3), *Trichuris vulpis* in 3.5% (16/457, 95% CI: 1.9–5.4), *Dipylidium caninum* in 2.2% (10/457, 95% CI: 0.07–3.2), *Eucoleus* spp. nematodes in 2% (8/457, 95% CI: 0.07–3.2), and fish-borne trematode eggs (*Opisthorchid/Heterophyid*) in 2% (9/457, 95% CI: 0.07–3.2) (Figure 2). Nearly one-third of dogs were infected by a single parasite group (32.6%, 95% CI: 28.6–36.9) and 12.1% (95% CI: 9.1–15.1) harboured mixed infections.

**Figure 3:**
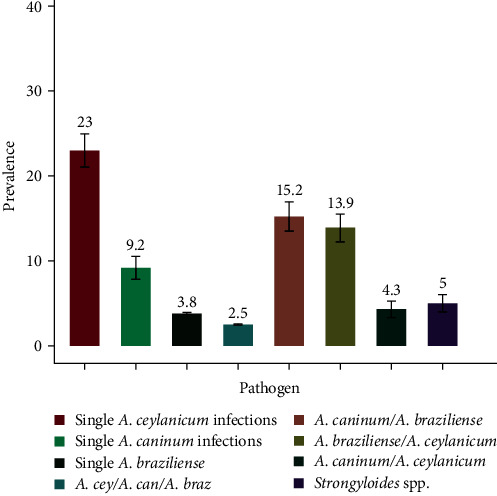
Prevalence of hookworms and *Strongyloides* spp. across five localities in Cambodia using multiplex qPCR. Error bars represent the standard error mean.

Overall, of the 447 samples for which DNA isolation was successful, 71.8% (321/447, 95% CI: 67.2–75.5) were positive for ≥1 hookworm species. Single *A. ceylanicum*, *A. caninum*, *and A. braziliense* infections accounted for 15.2% (68/447, 95% CI: 11.8–18.4), 13.9% (61/447, 95% CI: 10.4–16.7), and 4.3% (19/447, 95% CI: 2.4–6.1) of the hookworm infections, respectively. Mixed hookworm infections are reported in Figure 3. No *Uncinaria stenocephala* infections were detected. In addition, 5% (21/447, 95% CI: 2.7–6.2) of dogs were infected with *Strongyloides* spp.

**Figure 4:**
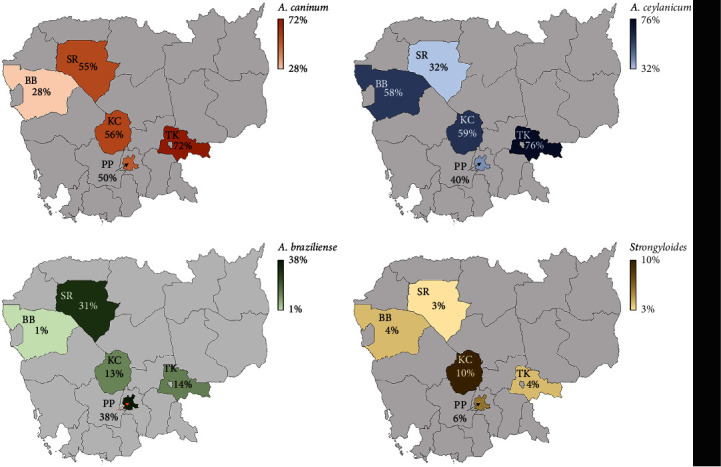
Prevalence of zoonotic hookworms and *Strongyloides* spp. infections per locality in Cambodia.

By locality, at an individual species level, *A*. *ceylanicum* infection prevalence ranged from 32% in PP to 76% in TB (*x* = 52.2, 95% CI: 32.5–74.2), *A. caninum* infections from 28% in BB to 72% in TB (*x* = 52.2, 95% CI: 32.5–71.8), while *A. braziliense* infections ranged from 1% in BB to 38% in PP (*x* = 19.4, 95% CI: 0.88–37.9). There was no statistical difference found between *A. ceylanicum* and *A. caninum* infections among locations, but there were significant mean differences between *A. braziliense* and *A. caninum* (*p* = 0.019)/*A. ceylanicum* (*p* = 0.023) infections among localities *Strongyloides* infections ranged from 3% in BB to 10% in KC with no statistical difference among locations (*x* = 5.4, 95% CI: 1.93–8.86) (Figure 4).

Using a combination of coproscopy and qPCR, the prevalence of hookworm increased from 25.1% to 73% (333/457, 95% CI: 68.8–76.9) and the overall prevalence of helminth infections from 44% to 79% (362/457, CI: 75.5–82.9). Of the dogs infected, 78% (360/457, 95% CI: 75–83) harboured at least one zoonotic helminth.

M-qPCR detected 46% (*n* = 206) more hookworm infections than coproscopy alone, with the difference being statistically significant (*p* < 0.0001). Kappa statistics demonstrated only slight agreement between qPCR and coproscopy for the detection of hookworm infections (ƙ = 0.162, 95% CI: 0.086–0.238). Twelve samples were coproscopy positive, but M-qPCR negative (Table 3).

### 3.2. cPCR and Sanger Sequencing

Of the 44 samples positive for *Spirometra* eggs, 15 were successfully amplified at the ITS-2 gene and one at the COX-1 gene. Of these, nine sequences showed 99%–100% nucleotide (nt) identity with sequences of *Spirometra erinaceieuropae* (GenBank accession numbers KC561781 and FJ886746–FJ886755 from cats and dogs, respectively). The COX-1 sequence showed 98.9% nt identity with *Spirometra mansoni* (GenBank accession number AB369251). Of the nine samples positive for opisthorchiid/heterophyid-like eggs, three were successfully characterised over a partial region of the ITS-2 gene, revealing a 99.6%–100% nt identity with sequences of *Haplorchis yokogawai* (GenBank accession number AB517589–AB517590). In addition, two samples showed 98.3%–99.59% nt identity with *Fischoederius elongatus*, one with *Paramphistomum* spp. with 99% nt identity (GenBank accession number MT268103) and two with *Gastrothylax crumenifer* with 99.59% nt identity (GenBank accession number KU530204). Three sequences showed 95.58%–96.6% nt identity with *Schistosoma* spp. (GenBank accession numbers MF776590.1, MG554659.1, OX103912.1 JQ289757.1, and HE601625.3) and therefore the lowest level taxonomic designation that could be given to these sequences was at the genus level (GenBank accession numbers S1 file). Of the nine samples positive for *Eucoleus*-like eggs, none could be characterised to a species level using a partial region of the COX-1 gene. Of the 67 *Toxocara* spp. positive infections by microscopy, all were attributed to *T. canis* (Table 4).

### 3.3. Age-Related Prevalence and Intensity of Hookworm and Toxocara Infections

**Figure 5:**
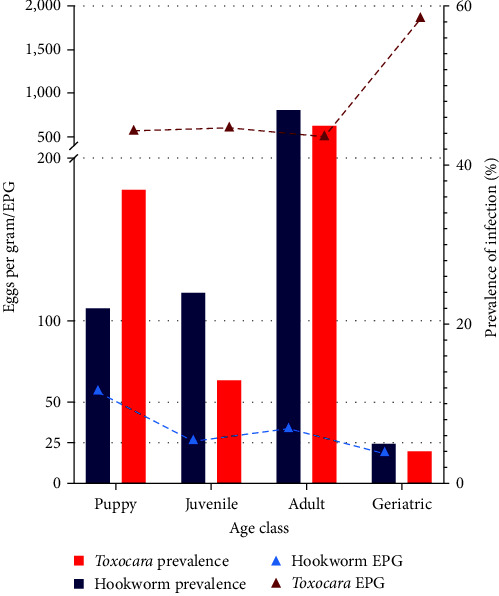
Prevalence and intensity (eggs per gram) of hookworm and *Toxocara canis* infections in dogs of different age categories by coproscopy.

The mean infection intensity of hookworm and *Toxocara* eggs was 37.5 EPG (95% CI: 22.9–52.1) and 567 EPG (95% CI: 369.9–764.4), respectively. The prevalence of hookworm and *Toxocara* peaked in adult dogs and was recorded at its lowest in geriatric dogs. Hookworm faecal egg-shedding intensity peaked in pups, however, no significant difference was observed among age classes and hookworm EPG, suggesting that the infection intensity means per age group were not statistically different from one another. *Toxocara* infection intensities were similar in puppies and adults, but exponentially increased in geriatric dogs (Figure 5). ANOVA analyses showed a significant mean difference between *Toxocara* EPG and age class (*p*-value 0.01, *F* = 4.01). Since the overall mean difference was significant for *Toxocara*, a post hoc test using Tukey's multiple comparison was conducted to compare the variation between each age class group (Table 5). The results indicated that dogs in the geriatric age class had significantly higher infection intensities than adults, juveniles, and puppies.

### 3.4. Risk Predictor Analyses

The multiple logistic regression model identified pale mucous membranes and a decrease in total protein as significant predictors (*p* < 0.05) of positivity to at least one GI helminth and hookworms (Table 6). For each increase in total protein, there is a 0.86 decrease in the odds of being infected with hookworms. Animals with pale mucous membranes were 1.96 times more likely to harbour hookworm infections.

## 4. Discussion

In this study, we report a high endemicity and diversity of intestinal zoonotic helminths affecting dogs across Cambodia. We emphasise the utility of integrating coproscopy and molecular approaches for the accurate identification of parasitic helminths and for the assessment of their prevalence, distribution, and risk predictors to ultimately improve their management through targeted control and prevention strategies.

Based on microscopy and molecular diagnostic approaches, zoonotic canine intestinal parasites were found highly endemic across all sampled locations. In agreement with previous studies, zoonotic hookworms were the most common helminths infecting Cambodian dogs [16, 17, 42]. However, given the resemblance of eggs of different hookworm species and the predominant use of microscopy in the above-mentioned studies, species level identification of all hookworms infecting dogs in Cambodia was missing. In this study, with the aid of molecular tools, we report for the first time, the presence of *A. braziliense* infections in Cambodia. Despite the endemic nature of *A. braziliense* in dogs, no cases of hookworm-related creeping eruptions have been reported in Cambodia to date. However, it is possible that these lesions may be misdiagnosed if medical practitioners in Cambodia are not aware of the aetiology and clinician characteristics of the disease caused by *A. braziliense*. Half of the dogs sampled harboured *A. ceylanicum* across all studied locations [16, 64, 65]. Data on the distribution of *A. ceylanicum* in canines can aid in identifying communities at risk of this zoonotic pathogen [23, 24, 52]. Endemic-hookworm infections in humans as identified by both microscopy and PCR, continue to be reported in Cambodia [52, 66, 67] with previous data demonstrating shared haplotypes of *A. ceylanicum* between dogs and humans [16, 23]. Active monitoring of this zoonotic hookworm in humans remains pertinent as ongoing Mass Drug Administration programmes targeting only humans, may have little impact on *A. ceylanicum* occurrence should dogs remain untreated and continue to contaminate the environment with infective larvae.

*Ancylostoma caninum* infections were also moderately to highly prevalent in the dogs sampled in this study. Despite only preliminary evidence on the ability of *A. caninum* to complete its life cycle in humans [68], *A. caninum* is a well-recognised aetiological agent of acute eosinophilic enteritis in people [24]. Although no cases have been reported in Cambodia thus far, the nonspecific nature of its symptoms makes it likely that this pathogen represents yet another underdiagnosed pathogen in the region [27, 29].

*Strongyloides* spp. was detected in canines in all locations sampled with a prevalence of 2%–10%, similar to previous reports in dogs from other Cambodian provinces (8%–14.9%) [17, 42], and a single study that found a much higher prevalence of 75.9%–88.63% in Preah Vihear province [30]. Although the zoonotic potential of canine *S. stercoralis* remains controversial, recent studies based on molecular phylogeny have provided strong evidence that dogs may act as reservoirs for the zoonotic transmission of *S. stercoralis* to humans in Cambodia [30], Thailand [69] and Myanmar [31]. Considering the high endemicity of *S. stercoralis* infections in humans, especially in school-aged children living in Cambodia [14, 30, 67], reporting its distribution and prevalence in dogs is important given continuing efforts to understand the zoonotic nature of this parasite and thereby improve its control.

*Toxocara canis* infections as confirmed by PCR and Sanger sequencing were detected in 15% of the dogs investigated by coproscopy, which to date, is the highest prevalence recorded in dogs in Cambodia (6.4%–8%) [17, 42]. Human toxocarosis is a globally neglected zoonosis categorised as a public health priority by the Centre for Disease Control and Prevention (CDC) [70, 71]. It is estimated that ≥100 million dogs are infected with *Toxocara* globally, shedding millions of eggs into the environment [37] and as a consequence, seroprevalence estimates show that 19% of humans are exposed to *Toxocara* worldwide [70, 72]. In SEA alone, the pooled prevalence of patent *T. canis* infection in dogs is 11.9% and the pooled seroprevalence in humans is estimated at 34.1% [37, 73]. In Cambodia, to our knowledge, neither epidemiological data nor case reports on human toxocarosis exist [73], making epidemiological surveys of this pathogen a research and health priority. In countries with similar ecoepidemiological conditions to Cambodia, such as Thailand, seroprevalence data reports that 6% of school-aged children [74] are exposed to *Toxocara*, in an area where *Toxocara* pooled prevalence is 5.4% in dogs [37]. Similarly, seroepidemiological studies in Vietnam have found that 30%–45% of people have been exposed to *Toxocara* spp. [75, 76]. Given the socioeconomical and climatic similarities between these countries, a similar prevalence could be expected in Cambodia.

In our study population, intensity measured as EPG of faeces demonstrated that dogs of all ages from puppies to geriatrics, contributed to the environmental contamination with hookworm eggs with no significant difference between age groups. However, in a recent study, dogs younger than 5 years and older than 15 years of age were more likely to be found infected with hookworms compared to 5–15-year-old dogs, with infection peaking in geriatric animals [8]. For *T. canis*, egg shedding intensities were similar in pups, juveniles, and adults, rising significantly in geriatric dogs. Although somewhat expected with hookworms [77], it is generally assumed that in dogs over the age of 6 months, patent egg-shedding infections with *T. canis* are uncommon, owing to a degree of age-related immunity that results in ingested larvae undergoing somatic, as opposed to tracheal migration [78]. This is supported by a number of epidemiologically studies of cared for household dogs [79–81]. However, this assumption has recently been challenged, demonstrating that repeated low infective doses of *T. canis* can consistently induce patency in adult dogs [82], especially those with challenged immunity [81, 83]. Geriatric dogs are likely to suffer greater immunosuppression than other age classes, owing to both infectious and non-infectious comorbidities (e.g., chronic ehrlichiosis, and neoplasia) that may exacerbate the rate and burden of patent *T. canis* infections in these communities [7, 84]. Thus, our results demonstrate that administration of anthelmintics in Cambodia should target dogs on mass, regardless of age, to minimise environmental contamination with hookworm and *T. canis* eggs. The multiple logistic regression model identified pale mucous membranes and low total protein as significant risk predictors for GI helminth and hookworm infections. Of these, hookworm infections are likely the main driver of this indicator. Hookworms are a well-known aetiological agent of haemorrhagic diarrhoea and melena that in turn, lead to anaemia and hypoalbuminemia [85, 86]. Because total protein were measured in this study, it is possible that the degree of hypoalbuminemia associated with hookworm infection in these dogs is grossly underestimated given the tick-borne pathogen-associated hyperglobulinemia found in the same cohort of dogs [84].

Dogs also play a role as reservoirs of zoonotic parasites that can be indirectly transmitted to humans through the ingestion of raw or undercooked crustaceans, fish, and reptiles [1, 87]. This study identified the tapeworms *S. erinaceieuropaei* and *S. mansoni* from our sampled dogs, both agents of human sparganosis [88–90]. Cases of sparganosis have been found in countries of SEA, such as Thailand and Vietnam [89, 91], with people showing symptoms ranging from asymptomatic to mild, for example, subcutaneous swelling, to severe, seizure, eosinophilia, and hemiparesis, depending on the site and size of lesions [92]. Additionally, we identified *Haplorchis yokogawai* a minute fish-borne intestinal trematode [87, 93] endemic to surrounding Thailand, Vietnam, and Laos. Although human cases have not yet been detected in Cambodia, a recent study revealed high levels of this parasite in freshwater fish in Phnom Penh and Pursat Province, suggesting a potential risk of infection to humans [87]. The presence of large trematode ova identified as *Schistosoma* spp. and species of amphistomes in dog faeces is not surprising, as dogs living in rural communities are likely to coprophage on faeces from a variety of hosts [94, 95]. Although dogs do not contribute to the direct transmission of these parasites, the misidentification of these eggs during coproscopic examination may lead to an incorrect diagnosis with regards to canines and their competence as biological hosts [63].

In alignment with other epidemiological surveys [15, 41, 45, 46, 96, 97], M-qPCR outperformed copromicroscopic diagnosis by detecting a significantly greater number of hookworm infections and allowed for differentiation of hookworm eggs to a species level. The M-qPCR also permitted the simultaneous detection of *Strongyloides* spp. without the need for subjecting fresh faecal samples to Koga-agar culture or the Baermann test. The vast discrepancy between test results reflected by only slightly agreement between coproscopy and qPCR for the detection of hookworm infections could be due to cold-chain interruption between sampling and analysis for a proportion of samples. Although the 5% w/v potassium dichromate-preserved faecal samples were kept on ice-refrigerated till analysis, power failures in Cambodia, and delays during air transportation (on ice) to Australia is likely to have resulted in embryonation and hatching of hookworm eggs precluding their detection by faecal floatation, therefore highlighting limitations of microscopy-based research.

In addition, cPCR-based genetic characterisation allowed parasite species such as *H. yokogawai*, *S. erinaceieuropaei*, and *S. mansoni* to be described for the first time in dogs from Cambodia, which would otherwise not have been achieved by coproscopy alone [15, 45, 46, 96, 97]. Chai et al. [47] addressed the issue of misdiagnosing small liver flukes such as *Opistorchis viverrini* using copro-examination only, which were in fact human infections attributed to *H. yokogawai*, *H. tachui*, and other small trematodes when using molecular tools. In addition, the use of molecular tools was crucial to report endemic *A. ceylanicum* infections in humans and dogs in the Americas and demonstrated that the geographical distribution of this parasite is not restricted to only Asia and Africa [54, 55].

Here, we provide a comprehensive picture of the distribution, prevalence, and risk factors of zoonotic intestinal helminths in dogs across Cambodia with the aid of copromicroscopic- and molecular-based diagnostic methods. Our data strongly recommend a “One-Health” approach to mitigating the zoonotic risks of these parasites through increased sanitation and hygiene and regular chemoprevention targeting dogs in mass, regardless of age and sex, in these disadvantaged communities.

## Figures and Tables

**Table 1:** Demographic data of the 457 dog samples assessed for gastroenteric parasites across five localities in Cambodia.

Study area	Sex		Age class (in years)				Husbandry		Neutered (*N*) suckling (*S*)
	Male	Female	Puppy <0.5	Juvenile >0.5–<1	Adult >1–<7	Geriatric >7	Owned	Community/pagoda	
Phnom Penh (PP) = 100	57	43	45	19	30	4	92	8	(*N* = 2), (*S* = 0)
Kampong Chhnang (KC) = 39	17	22	9	5	25	0	38	1	(*N* = 0), (*S* = 2)
Siem Reap (SR) = 125	80	45	20	22	76	5	16	109	(*N* = 2), (*S* = 2)
Battambang (BB) = 99	39	60	16	20	56	7	0	99	(*N* = 0), (*S* = 1)
Tbong Khmum (TK) = 94	43	51	16	37	33	5	0	94	(*N* = 0), (*S* = 0)
Total	232	214	106	103	220	21	146	311	*N* = 4, *S* = 5

Unreported data for age class and sex for 7 and 11 dogs, respectively.

**Table 2:** Gene targets, primer sequences, and thermocycling conditions of the qPCR assays used in this study for the detection of canine hookworms and *Strongyloides* spp.

Multiplex qPCR-target species	Primers and probes	Oligonucleotide sequence 5′-3′	qPCR conditions	Gene target	Size (bp)	Source
*A. ceylanicum* *A. caninum*	A. canceyF A. cancey R	GGG AAG GTT GGG AGT ATC G CGA ACT TCG CAC AGC AAT C	92°C/2 min ×1 95°C/10 s 60°C/60 s ×40	ITS-1	100	[51]
	AceyDOGCAT probe acantub probe	/Cy5/CCGTTC + CTGGGTGGC/3lAbRQSp /5HEX/AG + T+CGT + T + A + C + TGG/3IABkFQ				
*A. braziliense* *U. stenocephala*	UncbrazF Unbraz R	GAGCTTTAGACTTGATGAGCATTG GCAGATCATTAAGGTTTCCTGAC			119 118	
	Abra probe Unc probe	56FAM/TGAGCGCTA/ZEN/GGCTAACGCCT/3IABkFQ/ 5HEX/CATTAGGCG/ZEN/GCAACGTCTGGTG/3IABkFQ				
*Strongyloides* spp.	STR F STR R	CCAAGTAAACGTAAGTCATTAGC CGCCTCTGGATATTGCTCAGTTCC		18 S rRNA	101	[57]
	STR Probe	5Cy5/ACACACCGG/ZEN/CCGTCGCTGC/IBFQ				
*Canis lupus familiaris* DNA control	MAMF MAMR	CGACCTCGATGTTGGATCAG GAACTCAGATCACGTAGGACTTT		16 S MtrRNA	92	[49, 51]
	MAM probe	FAM/CCTAATGGT/ZEN/GCAGCAGCTATTAA/LABKFQ				
Equine herpes virus	EHV-FWD EHV-REV	GATGACACTAGCGACTTCGA TTTCGCGTGCCTCCTCCAG		Gb	81	[58]
	EHV probe	ROX/TTTCGCGTGCCTCCTCCAG/3IAbRQSp				

**Table 3:** Multiplex quantitative PCR (qPCR) and coproscopy agreement statistics.

Microscopy					
	qPCR	Positive	Negative	Total agreement (%)	Kappa (95% CI)
Hookworms	Positive	103	218	50	0.162 (0.086–0.238)
	Negative	12	124		

**Table 4:** Prevalence and distribution of faecal parasites detected in community dogs across five locations in Cambodia.

	Phnom Penh *n* = 100	Siem Reap *n* = 125	Kampong Chhnang *n* = 39	Battambang *n* = 99	Tbong Khmum *n* = 94	All locations *n* = 457
	(%) (95% CI)	(%) (95% CI)	(%) (95% CI)	(95% CI)	(95% CI)	(95% CI)
*Spirometra* spp. ^*∗*^ *Spirometra erinaceieuropaei*^Ŧ^ *Spirometra mansoni*^Ŧ^	3 (0.03–6.3) 0 0	3.2 (0–6) 0.4 (0–2) 0	36 (21–53) 10.5 (0.07–20) 2.6% (0–7.7)	13 (6–19) 2 (0–4) 0	11 (4–16) 3 0	9.6 (6.2–12.3)

Hookworms ^*∗*^ Hookworms^¥^ Hookworms ( ^*∗*^ + ^¥^combined)	25 (16.5–33.3) 68 (58.8–77.1) 69 (59.9–78.1)	24.8 (17.2–32.3) 58.2 (49.4–66.9) 62 (53.1–70.1)	33 (18.5–48.1) 81.6 (69.2–93.9) 82 (74–92.1)	10.1 (4.17–16) 65.3 (55.8–74.3) 65 (55.8–74.3)	10.6 (4.41–16.8) 94.6 (89.8–99.2) 95.7 (91.5–99.8)	25.1 (21.2–29.1) 71 (67.2–75.5) 73 (68.9–76.9)

*Ancylostoma ceylanicum*^¥^ *Ancylostoma caninum*^¥^ *Ancylostoma braziliense*^¥^	40 (30.4–49.6) 50 (40.2–59.8) 38 (28.5–47.5)	26 (18.4–34 45 (36.2–53.9) 25 (17.7–33.1)	60.5 (45–76.1) 58 (42.2–73.4) 13 (2.4–23.9)	58 (48.4–67.9) 29 (19.6–37.5) 1 (0–3)	77 (68.5–85.7) 74 (64.9–82.9) 14 (7–21.2)	50 (44.9–54.2) 50 (44.9–54.2) 20 (15.9–23.2)

*Toxocara canis* ^*∗*^^Ŧ^	17 (9.64–24.4)	21 (13.7–28)	8 (2–12.5)	7 (2–12)	15 (7–22)	15 (11.4–17.9)

*Trichuris vulpis* ^*∗*^	2 (0–4.7)	0.8 (0–4)	5 (0–12)	0	13 (6–19)	4 (1.9–5.4)

*Eucoleus* spp. ^*∗*^^Ŧ^	1 (0–5)	0.8 (0–4)	5 (0–12)	0	5 (0–9)	2 (0.07–3.24)

*Dipylidium caninum* ^*∗*^	3 (0.03–6.34)	2 (0–5)	2.5 (0–7)	2 (0–4)	2.1 (0–5)	2.2 (0.07–3.24)

Heterophyidae/opisthorchiidae-like eggs ^*∗*^ *Haplorchis yokogawai*^Ŧ^	1 (0–2.95) 0	2 (0–5) 0	2 (0–4) 0	8 (0–16.4 5 (0–12.3)	3.2 (0–6) 0.4 (0–2)	2.4 (1–3.8) 3

Large trematode eggs- mechanical ingestion *Fischoederius elongatus*^Ŧ*β*^ Paramphistomatidae spp.^Ŧ*β*^ *Gastrothylax crumenifer*^Ŧ*β*^ *Schistosoma* spp.^Ŧ*β*^	0 0 0 0 0	0 0 0 0 0	10.3 (8–15.5) 5.3 (2–8) 2.5 (0–5) 2.5 (0–5) 0	3 (0–5.5) 0 0 0 3 (0–5.5)	0 0 0 0 0	2.6 (0–5.5) 1.1 (0–2) 0.4 (0–2) 0.3 (0–1.5) 0.4 (0–2)

*Strongyloides* spp.^¥^	6 (1.35–10.6	3 (0–5.2)	10.5 (0.7–20)	2 (0–4)	3.2 (0–6)	5 (2.7–6.6)

Total prevalence	76 (67.3–84.4)	65.6 (57.3–73.4)	92 (84–100)	71 (62–79.7)	96 (91.7–99.8)	79 (75.5–82.9)

^*∗*^Microscopy, ^¥^M-qPCR, ^Ŧ^cPCR + sequencing, mechanical passage^*β*^.

**Table 5:** Age class and infection intensity comparison of absolute faecal egg counts for *Toxocara* and hookworms.

Source of variation	Sum of squares		Degrees of freedom		Mean sum of squares	*F* (Dfn, Dfd)		*p*-value
Hookworms								
Variation between age class EPG	10,120		3		3373	*F* (3,106) = 0.513		0.6741
Variation within age class EPG	696,723		106		6,573			
Total	706,842		109					
*Toxocara*								
Variation between age class EPG	7,135,955		3		2,378,652	*F* (3,61) = 4.09		^*∗*^0.0103
Variation within age class EPG	35,419,085		61		580,641			
Total	42,555,040		64					

**Tukey's multiple comparison between age classes**		**Mean difference**		**95% confidence interval**			**Adjusted *p*-value**	

Adults' vs. puppy		15.9		−558.2 to 588.6			0.999	
Adults' vs. geriatric		−1,338		−2,414 to −262.1			** ^*∗∗*^0.0090**	
Adults' vs. juvenile		125.7		−590.5 to 841.9			0.966	
Puppy vs. geriatric		−1,353		−2,447 to −259.1			** ^*∗∗*^0.0094**	
Puppy vs. juvenile		110.5		−632.7 to 853.7			0.979	
Geriatric vs. juvenile		1,464		288.5–2,639			** ^*∗∗*^0.0088**	

*α* = 0.05.  ^*∗∗*^*p* < 0.05.

**Table 6:** Clinical parameters estimates and odds ratios (95% profile likelihood) for positivity to at least one gastrointestinal parasite and hookworm in 457 dogs from Cambodia.

Parameter estimates	Estimate	Standard error	Odds ratios	*p*-value
At least one GI parasite				
Intercept	1.956	0.6,375		0.002
Total blood protein	−0.1,637	0.07,495	0.84 (0.73–0.98)	0.02
Pale mucous membranes	0.6,784	0.2,594	1.97 (1.17–3.26)	0.009

Hookworms				

Intercept	1.637	0.6,067		0.007
Total blood protein	−0.1,471	0.07,157	0.86 (0.75–0.99)	0.004
Pale mucous membranes	0.6,726	0.2,505	1.96 (1.2–3.2)	0.007

## Data Availability

The data that support the findings of this study are available from the corresponding author upon reasonable request.

## Supplementary Materials

Supplementary Materials GenBank submission accession numbers.
