# Supplementary material for: An Integrated Coproscopic and Molecular Method Provides Insights into the Epidemiology of Zoonotic Intestinal Helminths of Dogs across Cambodia
Source: Transbound Emerg Dis. 2023 Aug 22;2023:2001871. doi: 10.1155/2023/2001871 (PMC12016947; doi:10.1155/2023/2001871)
Supplement: Supplementary Materials — GenBank submission accession numbers. [file 2001871.f1.docx]

**Supplementary information**

**S1 file. GenBank submission accession numbers**

| **Species** | **Gene** | **GenBank Accession number** | **Number of sequences** |
| --- | --- | --- | --- |
| Fischoederius elongatus | ITS-2 | OQ459350, OQ459352 | 2 |
| Gastrothylax crumenifer | ITS-2 | OQ459351-OQ459354 | 2 |
| Schistosoma spp. | ITS-2 | OQ459347-OQ459349 | 3 |
| Paramphistomum spp. | ITS-2 | OQ459353 | 1 |
| Spirometra erinaceieuropaei | ITS-2 | OQ449427-OQ449435 | 9 |
| Spirometra mansoni | COX-1 | OQ442154 | 1 |
| Haplorchis yokogawai | ITS-2 | OQ443063-OQ443065 | 3 |
